# The impact of chronic intermittent hypoxia on enzymatic activity in memory-associated brain regions of male and female rats

**DOI:** 10.21203/rs.3.rs-5449794/v1

**Published:** 2024-12-13

**Authors:** Steve Mabry, Jessica L. Bradshaw, Jennifer J. Gardner, E. Nicole Wilson, Janak Sunuwar, Hannah Yeung, Sharad Shrestha, J. Thomas Cunningham, Rebecca L. Cunningham

**Affiliations:** University of North Texas Health Science Center; University of North Texas Health Science Center; University of North Texas Health Science Center; University of North Texas Health Science Center; University of North Texas Health Science Center; University of North Texas Health Science Center; University of North Texas Health Science Center; University of North Texas Health Science Center; University of North Texas Health Science Center

**Keywords:** calpain, caspase-3, chronic intermittent hypoxia, early growth response protein 1, sex differences

## Abstract

**Background:**

Obstructive sleep apnea (OSA) is an intermittent hypoxia disorder associated with cognitive dysfunction, including learning and memory impairments. There is evidence that alterations in protease activity and neuronal activation as associated with cognitive dysfunction, are dependent on sex, and may be brain region-specific. However, the mechanisms mediating OSA-induced cognitive impairments are unclear. Therefore, we used a rat model of OSA, chronic intermittent hypoxia (CIH), to investigate protease activity (e.g., calpain and caspase-3) and neuronal activation (early growth response protein 1, EGR-1) in brain regions associated with learning and memory. We used a rat model of OSA known as chronic intermittent hypoxia (CIH) to investigate protease activity (calpain and caspase-3) and neuronal activation (early growth response protein 1, EGR-1) in brain regions associated with learning and memory.

**Methods:**

Male and female Sprague Dawley rats were exposed to CIH or room air (normoxic) for 14 days. We quantified protease activity and cleaved spectrin products, along with EGR-1 protein expression in hippocampal subregions (CA1, CA3), cortical regions [entorhinal cortex (ETC), retrosplenial cortex (RSC), cerebellar cortex (CC)], and subcortical regions [raphe nucleus (RN), locus coeruleus (LC)] associated with learning and memory. Within each group, Pearson correlations of calpain activity, caspase-3 activity, and EGR-1 expression were performed between brain regions. Sex differences within normoxic and CIH correlations were examined.

**Results:**

CIH dysregulated calpain activity in male ETC and female CA1 and RSC. CIH dysregulated caspase-3 activity in male RN and female CA1 and RSC. CIH decreased calpain and caspase-3 cleavage products in male ETC. CIH decreased calpain-cleaved spectrin in male RSC but increased these products in female RSC. EGR-1 expression was decreased in male and female RN. Correlational analysis revealed CIH increased excitatory connections in males and increased inhibitory connections in females. EGR-1 expression in males shifted from negative to positive correlations.

**Conclusions:**

Overall, these data show that CIH dysregulates protease activity and impairs neuronal function in a brain region- and sex-dependent manner. This indicates that males and females exhibit sex-specific vulnerabilities to mild OSA. These findings concur with our previous behavioral studies that demonstrated memory impairment in CIH-exposed rats.

## Plain Language Summary

Many adults have a sleeping condition called sleep apnea, which can cause memory problems. Importantly, these memory problems can be linked to specific parts of the brain, such as the hippocampus, which is responsible for the formation of memories and ability to remember these memories. The hippocampus is connected to multiple other parts of the brain, so memory problems could also be due to changes in these other brain regions. Though sleep apnea is more common in men than women, it is likely that the brains of men and women respond to sleep apnea differently. To determine which brain regions are impacted by sleep apnea and how male or female sex could alter this effect, we exposed adult male and female rats to chronic intermittent hypoxia (CIH), which is a rat model of sleep apnea. We measured the effect of CIH by looking at the activation of brain cells and proteases, which are specialized proteins that cut other proteins into smaller pieces to change how they work. We found that CIH effects were unique to specific parts of the brain, and these effects were mainly observed in female rats. CIH also changed the way that enzyme and brain cell activity in certain brain regions was related to other brain regions. These results mean that not only does sleep apnea change the way parts of the brain function, but these changes are different for males and females. This indicates that men and women could experience sex-specific memory problems from sleep apnea.

## Background

Cognitive impairments are associated with many common sleeping disorders, such as obstructive sleep apnea (OSA) [1–7]. Approximately 10–26% of adults worldwide are diagnosed with OSA [8–10]. Structural alterations in the brain have been observed in patients with OSA [11–14]. These studies have identified brain region-dependent vulnerabilities to OSA, which notably included the hippocampus [11–14]. The hippocampus is a highly connected structure within the brain (Supplemental Fig. S1), and hippocampal dysfunction is routinely associated with cognitive impairments [15–17]. The hippocampus is separated into dorsal and ventral regions, with the dorsal hippocampus predominantly associated with learning and memory [18–20]. Within the dorsal hippocampus, the CA1 and CA3 subregions exhibit strong bidirectional connectivity [21, 22]. These regions also have strong efferent projections to brain regions, such as the entorhinal cortex (ETC) [15, 21, 23] and retrosplenial cortex (RSC) [23, 24]. The ETC is the primary afferent brain region to the hippocampus, and it is critical for processing much of the sensory information required to form memories [25–27]. The RSC, which is associated with spatial and working memory [24], also has bidirectional connections with the ETC [28, 29]. Hippocampal function is also directly regulated through the release of neuromodulatory neurotransmitters from the raphe nucleus (RN) [30] and locus coeruleus (LC) [31]. Hippocampal cognitive functions are also indirectly affected by cortical brain regions such as the cerebellar cortex (CC). The CC is associated with spatial navigation, motor coordination, and learning, and the CC signals to the hippocampus through the ETC and RN [32]. Though many brain regions are involved in hippocampal cognitive functions, little is known about how OSA affects most of these regions.

A major physiological consequence of untreated OSA is oxidative stress [33–36]. Protease enzymes (e.g., calpain, caspase-3) are critical to proper cellular function within the brain, and these enzymes are regulated by oxidative stress [37–39]. Calpain is integral to memory retrieval and consolidation by modulating neuronal activity [40–42], and calpain activity is observed throughout the healthy human brain [43]. Caspase-3 is classically associated with the initiation of apoptotic cascades [44, 45]; however, a growing body of evidence indicates that caspase-3 activity is also associated with increased cellular proliferation and survival [46–48]. Indeed, deficits in caspase-3 have been linked with neuronal dysfunction [49], decreased neurogenesis [50], and impaired memory consolidation [51]. Calpain activity may also regulate caspase-3, as calpain has been observed to suppress apoptosis associated with caspase-3 [38]. In addition to calpain and caspase-3, early growth response protein 1 (EGR-1) has been associated with apoptotic cascades [52], either inhibiting [53, 54] or inducing apoptosis [54, 55]. EGR-1 is a transcription factor expressed throughout the brain and is associated with neuronal and synaptic activity [56, 57]. Decreased EGR-1 expression in the hippocampus has been associated with cognitive impairments [56, 58]. Further, activity of EGR-1 has been observed to be dependent on both sex and brain region [59–62].

Chronic intermittent hypoxia (CIH) is an experimental rodent model for the hypoxemia associated with OSA. Fragmented hypoxic episodes [63], elevated inflammation [64–66], and cognitive impairments [66–69] observed in OSA are replicated through CIH exposure in rodents. Further, the effects of CIH have been observed to be brain region dependent [70–73]. Within the hippocampus, CIH can induce neuronal apoptosis and cognitive impairment [70–73]. CIH has also been linked to alterations in hippocampal protease activity [74]. Our group has observed brain region-dependent effects of CIH following 7 days of CIH exposure, where CIH decreased calpain activity in the ETC in male rats [75]. The effects of CIH on protease activity in specific brain regions in female rats has yet to be examined.

The goal of this study was to determine which brain regions were vulnerable to CIH-induced neuronal dysfunction. Additionally, as sex has been observed to affect both OSA severity [76, 77] and the CIH-induced behavioral and physiological phenotype [66], we examined if CIH-induced neuronal dysfunction was dependent on sex. To examine these effects, we measured calpain activity, caspase-3 activity, and EGR-1 expression in hippocampal subregions and extrahippocampal brain regions associated with hippocampal cognitive functions in male and female rats (Supplemental Fig. S1). These regions were selected based on cognitive impairments observed following 14 days of CIH exposure, which included deficits in recollective memory [66].

## Methods

### Animals

This experiment was part of a larger project, which examined the effects of CIH on inflammation, oxidative stress, and behavioral function [66]. All experiments were conducted using adult virgin Sprague Dawley male and female rats (aged 3–4 months, Charles River, Wilmington, MA). Male and female rats were single-housed in separate rooms in our animal facility on a 12-hour (hr) reverse light cycle (lights were off at 09:00). Food and water were provided *ad libitum*. Rats of each sex (n = 12/sex) were randomly assigned via simple randomization to either normoxia (room air) or CIH treatment conditions (n = 6/group). At the conclusion of the CIH protocol, the rats were anesthetized with 2–3% isoflurane and euthanized via decapitation during the active phase of the circadian cycle (09:00–11:00) [63, 64, 75, 78]. All experiments were conducted in agreement with the Guide for the Care and Use of Laboratory Animals of the National Institutes of Health and the ARRIVE guidelines. These protocols were approved by the Institutional Animal Care and Use Committee of the University of North Texas Health Science Center.

### Chronic intermittent hypoxia protocol

One week prior to the initiation of the CIH protocol, the home cages (clear plastic containers) of the rats were placed into Oxycycler chambers (76.2 × 50.8 × 50.8cm, BioSpherix, Lacona, NY, USA) to acclimatize the rats to the chambers under normoxic conditions. During their sleep phase of the circadian cycle, CIH was performed for 8 hrs starting at 21:00. The CIH protocol consisted of intermittent oxygen reduction from 21% (room air) to 10% in 6-minute cycles per hour (i.e., 10 cycles/hr) over 8 hrs/day for a period of 14 days, as previously described [63, 64, 78–80]. Cycling CIH 10 times per hour results in an apnea/hypopnea index of 10, which is consistent with mild sleep apnea in humans [79, 81].

### Sample collection

To collect tissue samples, brains were quickly removed, ash frozen in 2-methlylbutane, and sliced into 1-mm coronal sections using a brain matrix (RBM-4000C, ASI Instruments, Warren, MI). The following brain regions were isolated based on mm from Bregma according to Paxinos and Watson’s brain atlas [82] using blunt 20-guage needles attached to 1 mL syringes for brain region microdissection [63, 64, 75, 78]: CA1 region of the dorsal hippocampus (CA1, −5.30 mm); CA3 region of the dorsal hippocampus (CA3, −5.30 mm); entorhinal cortex (ETC, −5.30 mm); retrosplenial cortex (RSC, −5.30 mm); raphe nucleus (RN, −7.64 mm); locus coeruleus (LC, −9.68 mm); cerebellar cortex (CC, −10.04 mm). Micro-dissected brain samples were placed into microcentrifuge tubes to be stored at −80°C until protein analysis.

### Tissue sample preparation, electrophoresis, and western blots

Tissue sample preparation, electrophoresis, and western blots

For protein analysis, frozen tissue samples were thawed in RIPA buffer (VWR, cat #N653) containing (per 0.5 ml): 2.5 μl Halt^™^ protease and phosphatase inhibitor (Thermo Scientific, cat #78442), 1 μl 0.5 M ethylenediaminetetraacetic acid (EDTA, Sigma-Aldrich), and 1 μl 0.5 mM dithiothreitol (DTT, Sigma-Aldrich) for homogenization, as previously described [63, 78, 83–87]. Total protein concentration levels in the homogenate were determined using Pierce BCA Protein Assay (Thermo Fisher, cat #23225). Samples were denatured with β-mercaptoethanol and boiled at 100°C for 5 min. Equal volumes of denatured tissue samples containing 20 μg protein were loaded into a Bio-Rad 4–15% polyacrylamide gel. The gel then underwent electrophoresis at 80 milliamps (mA) in a tris-glycine running buffer followed by 10 min rapid transfer using a Bio-Rad Trans-Blot^®^ Turbo Transfer System. Following 30 minutes washing, membranes were blocked for 30 minutes with 5% nonfat milk in tris-based saline (TBS)-0.1% Tween 20 (TBST) at room temperature. Membranes were then transferred to 1% nonfat milk TBST solutions containing specific primary antibodies and incubated overnight at 4°C. Primary antibodies against early growth response protein 1 (EGR-1; Santa Cruz, sc-51830, 1:100) and spectrin (Millipore Sigma, MAB1622, 1:1000) were applied on all membranes. Afterwards, membranes were washed in 10-minute increments for 30 minutes, and then incubated in 1% milk TBST-secondary antibody solutions including HRP-conjugated horse anti-mouse (Cell Signaling, 7076P2, 1:2500) or HRP-conjugated goat anti-rabbit (Cell Signaling, 7074P2, 1:2500) at room temperature for 1 hour. For protein normalization, we used β-actin primary antibody (GeneTex, GTX 629630, 1:3000), which was incubated for 1 hour at room temperature. Protein bands were visualized using ECL^™^ Prime Western Blotting Detection Reagents (Cytiva, RPN2232) and enhanced chemiluminescence detection in a Syngene G:Box system using GeneSys Image Acquisition software (Syngene, version 1.5.2.0) as previously described [63, 78, 86]. NIH Image J software (version 1.48v) was used to quantify band densitometry of proteins of interest normalized to β actin band densitometry. EGR-1 (54–58 kD) was measured as a marker of neuronal activation [88, 89]. Spectrin is a cytoskeletal membrane protein associated with neurotransmitter release in the brain [38, 90–92]. Uncleaved spectrin (250 kD) and enzymatic cleavage of spectrin by calpain (150 kD) and caspase-3 (120 kD) were measured. Calpain and caspase-3 activity were quantified as a percentage of the measured cleavage product compared to uncleaved spectrin. Activity indicates how much cleavage each enzyme is performing (i.e., calpain, caspase-3) [75, 86]. EGR-1 and spectrin cleavage products were measured as % of β-actin expression. Expression of calpain and caspase-3 cleaved spectrin indicates the cellular concentration of cleaved spectrin, and provides information to the functional state of the cell, as these cleaved products are associated with altered cellular signaling [40, 43, 91]. Representative images of Western blot membranes can be found in Supplemental Fig. S2.

### Statistical analysis: Protein expression

Statistical analyses were conducted in IBM^®^ SPSS^®^ (SPSS^®^ v. 29.0.0, IBM^®^, 2022). Normality of data distribution was tested using the Shapiro-Wilk test. Outliers were identified by Tukey’s method in SPSS and excluded from analysis. 2-way ANOVAs were conducted using the factors of CIH and sex. For all significant effects, we provide the F values, degrees of freedom, p-values, and η^2^ (measure of effect size) in the figure legend. Following ANOVA testing, a Fisher’s LSD post hoc test was used to determine specific group differences for significant results. Results are presented as mean ± S.E.M. Results were graphed with GraphPad Prism (v. 10.2.1, © 2024 GraphPad Software). Significance was defined as p ≤ 0.05.

### Statistical analysis: Correlation heatmaps and connectomes

Following methodology described by Yagi et al. (2022), correlation heatmaps and connectomes were generated to examine if protease activity or neuronal activity are associated within connected brain regions, and if these relationships are associated with sex or CIH exposure [62]. The overall analysis and visualization were conducted using various open-source scientific packages, including Jupyter Notebook (v. 6.5.4) [93], Matplotlib (v. 3.8.0) [94], Numpy (v. 1.26.4) [95], Pandas (v. 2.1.4) [96], and Seaborn (v. 0.12.2) [97]. Connectomes for males and females were created using a python library package networkx (v. 3.1) [98] using the Pearson Correlation Coefficient [99] matrix of the different brain regions. A gradient color scale is used to indicate the strength and direction of the Pearson correlation, with dark red indicating strong positive correlations, and dark blue indicating strong negative correlations. Combined heatmaps containing both males and females were generated with correlations in opposite directions (sex differences) highlighted with a green border and a circle. Fisher’s z-test statistic was used to determine significance in these sex differences. Each node in the connectome represents distinct brain regions. Darkness of color and thickness of network edges represent the strength of the correlation. Correlations larger than ± 0.67 are labeled on the connectomes. Note that a robust ‘min_periods = 1’, ignoring missing values approach was taken to appropriately handle missing values within the dataset to ensure data integrity and avoid the effects of data imputations. Significance for Pearson correlations and z-test statistics was defined as p < 0.10.

## Results

### CIH induced sex-dependent dysregulation of protease activity in the hippocampus and cortical regions

**Figure 1:**
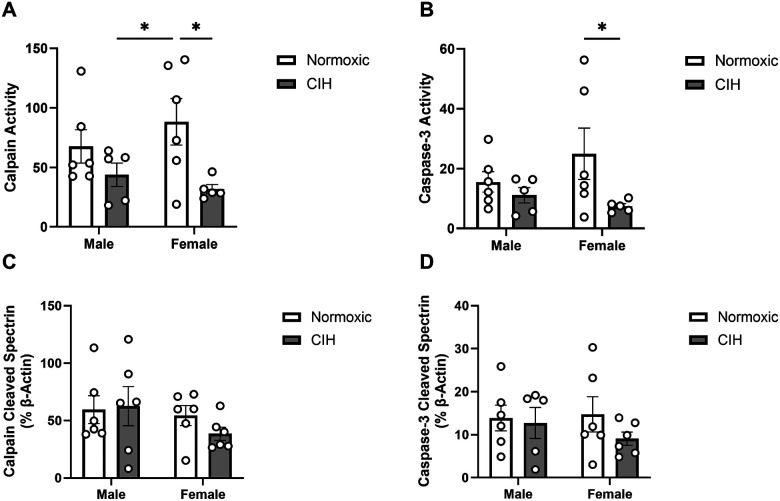
Protease activity and cleavage products in the CA1 **(A)** Quantified calpain activity, **(B)** quantified caspase-3 activity, **(C)** expression of calpain cleaved spectrin, and **(D)** expression of caspase-3 cleaved spectrin in the CA1. Calpain and caspase-3 activity represent % of protease cleaved spectrin to uncleaved spectrin (250 kD). Calpain cleaved spectrin (150 kD) and caspase-3 cleaved spectrin (120 kD) expression normalized to β-actin. Raw values are shown and error bars denote mean ± S.E.M. Analyzed by 2-way ANOVA with Fisher’s LSD multiple comparisons tests. Post-hoc significance indicated by: * = p≤0.05. Significant effects observed: **(A)**: CIH (F_1, 18_=8.100; p=0.011; η^2^=0.294); **(B)**: CIH (F_1, 18_=4.407; p=0.050; η^2^=0.182). *CIH: Chronic intermittent hypoxia*.

CIH decreased calpain activity in the CA1 (p = 0.011; Fig. 1A), primarily in female rats. Caspase-3 activity was similarly decreased by CIH in the CA1 (p = 0.050; Fig. 1B), primarily in female rats. However, there were no CIH effects on spectrin cleavage products in the CA1 (Fig. 1C, D). Moreover, no effects of sex were observed on calpain or caspase-3 activity in the CA1 (Fig. 1). No effects of CIH or sex were observed on calpain activity, caspase-3 activity, or spectrin cleavage products in the CA3 (Table 1).

**Figure 2:**
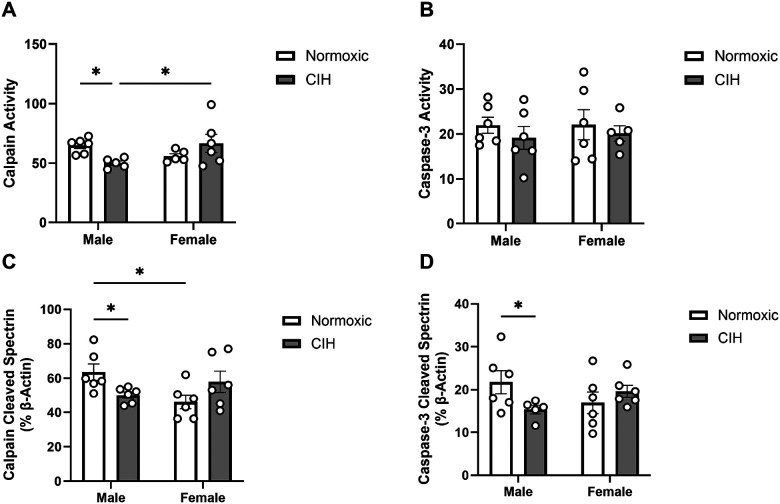
Protease activity and cleavage products in the entorhinal cortex **(A)** Quantified calpain activity, **(B)** quantified caspase-3 activity, **(C)** expression of calpain cleaved spectrin, and **(D)** expression of caspase-3 cleaved spectrin in the ETC. Calpain and caspase-3 activity represent % of protease cleaved spectrin to uncleaved spectrin (250 kD). Calpain cleaved spectrin (150 kD) and caspase-3 cleaved spectrin (120 kD) expression normalized to β-actin. Raw values are shown and error bars denote mean ± S.E.M. Analyzed by 2-way ANOVA with Fisher’s LSD multiple comparisons tests. Post-hoc significance indicated by: * = p≤0.05. Significant effects observed: **(A)**: CIH X Sex (F_1,18_=7.565; p=0.013; η^2^=0.286); **(C)**: CIH X Sex (F_1, 20_=7.891; p=0.010; η^2^=0.274); **(D)**: CIH X Sex (F_1, 19_=4.635; p=0.044; η^2^=0.190). *CIH: Chronic intermittent hypoxia; ETC: Entorhinal cortex*.

An interaction between CIH and sex was observed on calpain activity in the ETC (p = 0.013; Fig. 2A), where CIH decreased calpain activity only in males. No effect of CIH or sex was observed on caspase-3 activity in the ETC (Fig. 2B). Similar to calpain activity, an interaction between CIH and sex was observed on calpain cleaved spectrin protein expression (p = 0.010; Fig. 2C) and was reduced by CIH only in males. Unlike caspase-3 activity in the ETC, an interaction between CIH and sex was observed on caspase-3 cleaved spectrin protein expression (p = 0.044; Fig. 2D), which was also reduced by CIH only in males.

**Figure 3:**
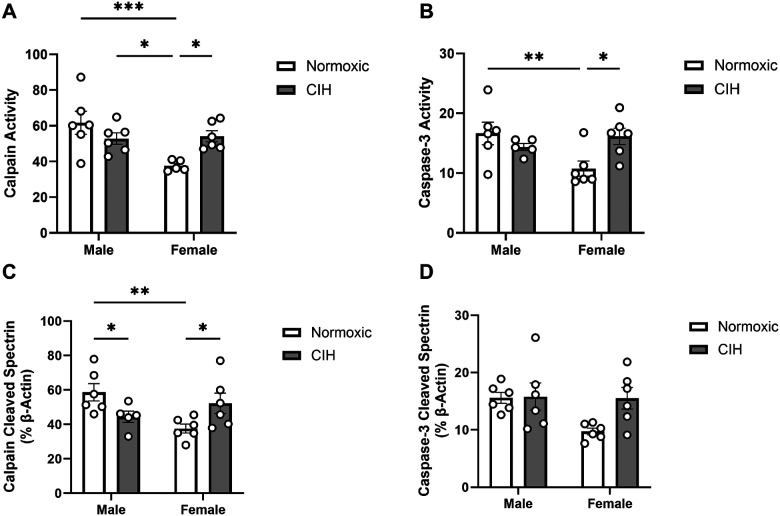
Protease activity and cleavage products in the retrosplenial cortex **(A)** Quantified calpain activity, **(B)** quantified caspase-3 activity, **(C)** expression of calpain cleaved spectrin, and **(D)** expression of caspase-3 cleaved spectrin in the RSC. Calpain and caspase-3 activity represent % of protease cleaved spectrin to uncleaved spectrin (250 kD). Calpain cleaved spectrin (150 kD) and caspase-3 cleaved spectrin (120 kD) expression normalized to β-actin. Raw values are shown and error bars denote mean ± S.E.M. Analyzed by 2-way ANOVA with Fisher’s LSD multiple comparisons tests. Post-hoc significance indicated by: * = p≤0.05; ** = p≤0.01; *** = p≤0.001. Significant effects observed: **(A)**: Sex (F_1, 19_=7.443; p=0.013; η^2^=0.204), CIH X Sex (F_1, 19_=9.216; p=0.007; η^2^=0.253); **(B)**: CIH X Sex (F_1, 19_=7.367; p=0.014; η^2^=0.248); **(C)**: CIH X Sex (F_1, 19_=10.110; p=0.005; η^2^=0.324). *CIH: Chronic intermittent hypoxia; RSC: Retrosplenial cortex*.

In the RSC, effects of sex (p = 0.013) and an interaction between CIH and sex (p = 0.007) were observed on calpain activity (Fig. 3A). Specifically, normoxic females had lower calpain activity than normoxic and CIH males, and CIH increased calpain activity only in females. Similarly, an interaction between CIH and sex was observed on caspase-3 activity in the RSC (p = 0.014; Fig. 3B), with lower caspase-3 activity observed in normoxic females than normoxic males, and caspase-3 activity increased by CIH only in females. An interaction between CIH and sex was observed on calpain cleaved spectrin protein expression in the RSC (p = 0.005; Fig. 3C). Similar to calpain activity in the RSC, normoxic females had less calpain cleaved spectrin protein expression than normoxic males, and CIH increased calpain cleaved spectrin protein expression in females. However, unlike calpain activity in the RSC, CIH also decreased calpain cleaved spectrin protein expression in males. No effects of CIH or sex were observed on caspase-3 cleaved spectrin protein expression in the RSC (Fig. 3D). No effects of CIH or sex were observed on calpain activity, caspase-3 activity, or protein expression of spectrin cleavage products in the CC (Table 1).

### CIH had limited effects on protease activity in regions which modulate hippocampal cognitive function

**Figure 4:**
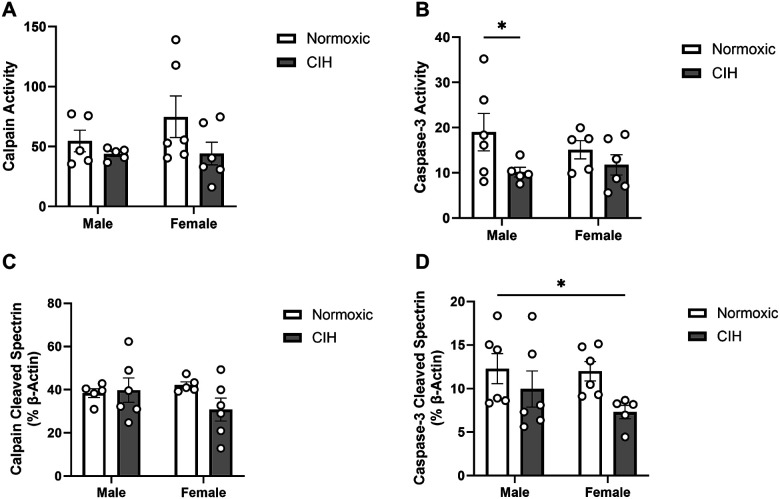
Protease activity and cleavage products in the raphe nucleus **(A)** Quantified calpain activity, **(B)** quantified caspase-3 activity, **(C)** expression of calpain cleaved spectrin, and **(D)** expression of caspase-3 cleaved spectrin in the RN. Calpain and caspase-3 activity represent % of protease cleaved spectrin to uncleaved spectrin (250 kD). Calpain cleaved spectrin (150 kD) and caspase-3 cleaved spectrin (120 kD) expression normalized to β-actin. Raw values are shown and error bars denote mean ± S.E.M. Analyzed by 2-way ANOVA with Fisher’s LSD multiple comparisons tests. Post-hoc significance indicated by: * = p≤0.05; ** = p≤0.01. Significant effects observed: (B): CIH (F_1, 18_=4.758; p=0.043; η^2^=0.199); **(D)**: CIH (F_1, 19_=5.036; p=0.037; η^2^=0.198). CIH: Chronic intermittent hypoxia; RN: Raphe nucleus.

Calpain activity in the RN was not affected by CIH (Fig. 4A). CIH decreased caspase-3 activity in the RN (p = 0.043; Fig. 4B), particularly in males. No effects of CIH were observed on calpain cleaved spectrin protein expression in the RN (Fig. 4C). CIH also decreased caspase-3 cleaved spectrin protein expression in the RN (p = 0.037; Fig. 4D), specifically in females. No effects of sex were observed on calpain activity, caspase-3 activity, or protein expression of spectrin cleavage products in the RN (Fig. 4). No effects of sex or CIH were observed on calpain activity or calpain cleaved spectrin protein expression in the LC (Table 1). Caspase-3 cleaved spectrin protein expression was not observed in the LC.

### CIH only decreased neuronal activity in the raphe nucleus

**Figure 5:**
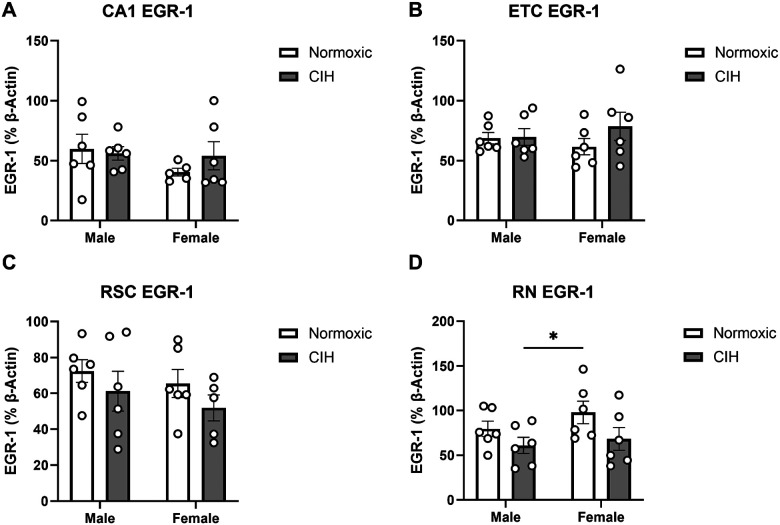
EGR-1 expression in the hippocampus and associated brain regions **(A)** Quantified EGR-1 expression in the CA1; **(B)** ETC; **(C)** RSC; and **(D)** RN. EGR-1 expression normalized to β-actin. Raw values are shown and error bars denote mean ± S.E.M. Analyzed by 2-way ANOVA with Fisher’s LSD multiple comparisons tests. Post-hoc significance indicated by: * = p≤0.05. Significant effect observed **(D)**: CIH: (F_1, 20_=4.857; p=0.039; η^2^=0.183). *CIH: Chronic intermittent hypoxia; ETC: Entorhinal cortex; RN: Raphe nucleus; RSC: Retrosplenial cortex*.

EGR-1 is a transcription factor associated with cognitive function and neuronal activity [56–58]. Though CIH dysregulated protease activity in multiple brain regions, EGR-1 protein expression was unaffected in most brain regions. Despite protease activity dysregulation, no effects of CIH or sex were observed on EGR-1 expression in the CA1 (Fig. 5A), ETC (Fig. 5B), or RSC (Fig. 5C). However, CIH decreased EGR-1 protein expression in the RN (p = 0.039; Fig. 5D). No effect of sex was observed on EGR-1 protein expression in the RN (Fig. 5D). No effects of CIH or sex were observed on EGR-1 protein expression in the CA3, CC, or LC (Table 2).

### CIH modulates sex-dependent patterns of inter-regional associations in protease activity

**Figure 6:**
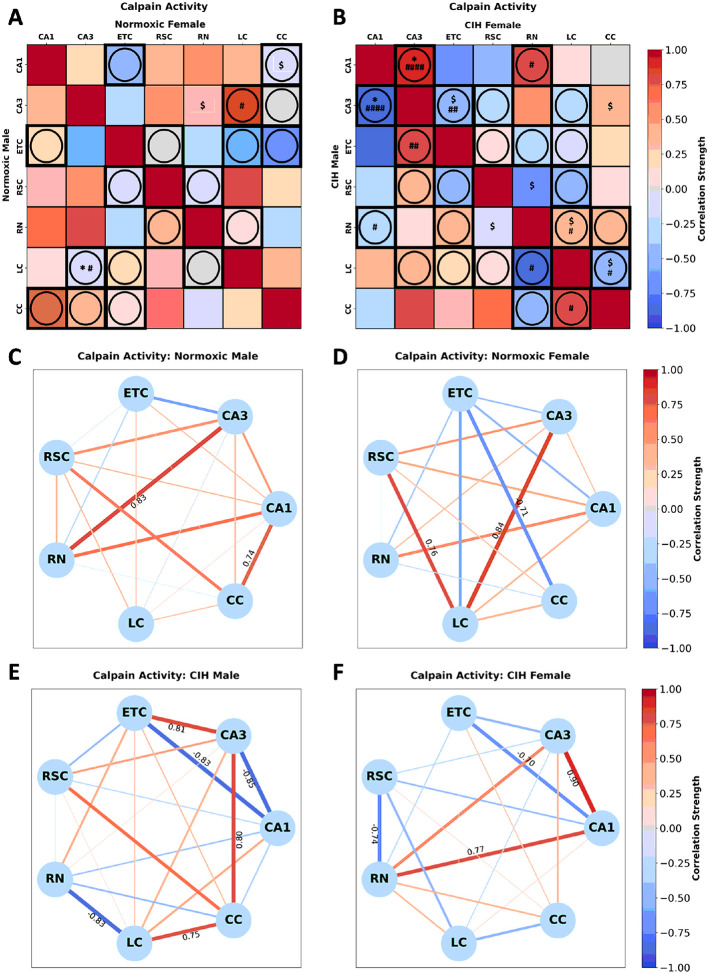
Sex and CIH differences in the inter-regional correlations of calpain activity **(A)** Heatmaps of calpain activity in normoxic and **(B)** and CIH males and females. **(C)** Correlation connectomes for normoxic males, **(D)**normoxic females, **(E)** CIH males, and **(F)** CIH females. Darkness of color **(A-F)** and thickness of network edges **(C-F)** represent the strength of the correlation, with correlations larger than ± 0.67 labeled. Correlations between brain regions were analyzed by Pearson correlations. Significance indicated by: $ = p≤0.10; * = p≤0.05. Correlations that are in opposite directions are indicated with a circle and green outline. Significance between sexes was determine using z-test statistics. Significance indicated by # = p≤0.10; ## = p≤0.05; #### = p≤0.001.

Calpain activity in normoxic rats (Fig. 6A) was significantly correlated in males between LC-CA3 [r(n = 6) = −0.157, p = 0.036] and in females between CA1-CC [r(n = 6) = −0.059, p = 0.092] and CA3-RN [r(n = 6) = 0.29, p = 0.085]. Sex differences in calpain activity between CA3-LC (p = 0.090) were observed in normoxic rats (Fig. 6A). Conversely, calpain activity in CIH rats (Fig. 6B) was significantly correlated in males between CA3-CA1 [r(n = 5) = −0.846, p = 0.039] and RN-RSC [r(n = 5) = −0.17, p = 0.094] and in females between CA1-CA3 [r(n = 5) = 0.897, p = 0.039], CA3-ETC [r(n = 6) = −0.492, p = 0.100], CA3-CC [r(n = 6) = 0.393, p = 0.057], RSC-RN [r(n = 6) = −0.738, p = 0.094], RN-LC [r(n = 6) = 0.343, p = 0.079], and LC-CC [r(n = 6) = −0.519, p = 0.084]. Sex differences in calpain activity between CA1-CA3 (p = 0.001), CA1-RN (p = 0.087), CA3-ETC (p = 0.043), RN-LC (p = 0.056), and LC-CC (p = 0.057) were observed in CIH rats (Fig. 6B). As can be observed in Fig. 6C and Fig. 6D, few strong correlations in calpain activity are present in normoxic males and females. Strong positive correlations between CA3-RN and CA1-CC were observed in normoxic males, whereas strong positive correlations between CA3-LC and RSC-LC and a strong negative correlation between ETC-CC were observed in normoxic females. As can be observed in Fig. 6E and 6F, CIH changes the strength and direction of many correlations in calpain activity in both males and females. In CIH males, strong positive correlations between CA3-ETC, CA3-CC, and LC-CC, and strong negative correlations between CA1-CA3, CA1-ETC, and RN-LC were observed. Conversely, strong positive correlations between CA1-CA3 and CA1-RN, and strong negative correlations between RSC-RN and CA1-ETC were observed in CIH females.

**Figure 7:**
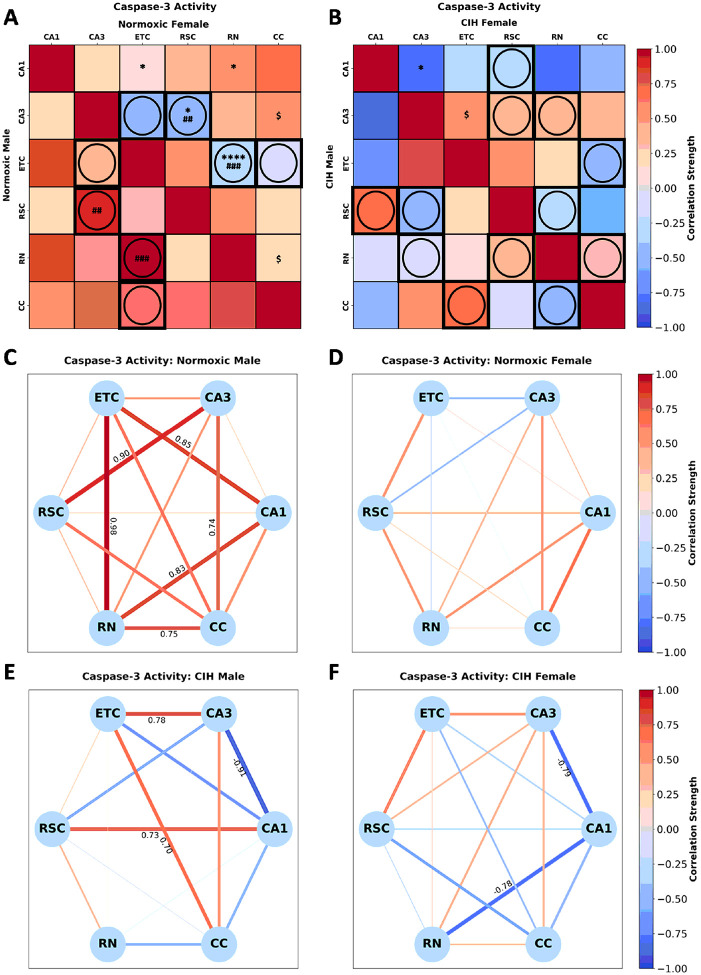
Sex and CIH differences in the inter-regional correlations of caspase-3 activity **(A)** Heatmaps of caspase-3 activity in normoxic and **(B)** and CIH males and females. **(C)** Correlation connectomes for normoxic males, **(D)**normoxic females, **(E)** CIH males, and **(F)** CIH females. Darkness of color **(A-F)** and thickness of network edges **(C-F)** represent the strength of the correlation, with correlations larger than ± 0.67 labeled. Correlations between brain regions were analyzed by Pearson correlations. Significance indicated by: $ = p≤0.10; * = p≤0.05; **** = p≤0.001. Correlations that are in opposite directions are indicated with a circle and green outline. Significance between sexes was determine using z-test statistics. Significance indicated by ## = p≤0.05; ### = p≤0.01.

Caspase-3 activity in normoxic rats (Fig. 7A) was significantly correlated in females between CA1-ETC [r(n = 6) = 0.138, p = 0.030], CA1-RN [r(n = 5) = 0.512, p = 0.041], CA3-RSC [r(n = 6) = −0.399, p = 0.014], CA3-CC [r(n = 6) = 0.505, p = 0.092], ETC-RN [r(n = 5) = −0.233, p < 0.001] and RN-CC [r(n = 5) = 0.212, p = 0.085]. No significant correlations in caspase-3 activity were observed in normoxic males. Sex differences in caspase-3 activity between CA3-RSC (p = 0.020), and ETC-RN (p = 0.003) were observed in normoxic rats (Fig. 7A). Conversely, caspase-3 activity in CIH rats (Fig. 7B) was significantly correlated in females between CA1-CA3 [r(n = 5) = −0.795, p = 0.034], CA3-ETC [r(n = 5) = 0.549, p = 0.068]. No significant correlations in caspase-3 activity were observed in CIH males. No significant sex differences in caspase-3 activity were observed in CIH rats (Fig. 7B). As can be observed in Fig. 7C and Fig. 7D, multiple strong correlations in caspase-3 activity are present in normoxic males but not normoxic females. Strong positive correlations between CA1-ETC, CA1-RN, CA3-RSC, CA3-CC, ETC-RN, and RN-CC were observed in normoxic males, whereas no strong correlations were observed in normoxic females. As can be observed in Fig. 7E and 7F, CIH changes the strength and direction of many correlations in caspase-3 activity in both males and females. In CIH males, strong positive correlations between CA1-RSC, CA3-ETC, and ETC-CC, and a strong negative correlation was observed between CA1-CA3. Interestingly, strong negative correlations between CA1-CA3 and CA1-RN not observed in the normoxic females were observed in CIH females.

### CIH alters sex-dependent inter-regional associations in EGR-1 expression

**Figure 8:**
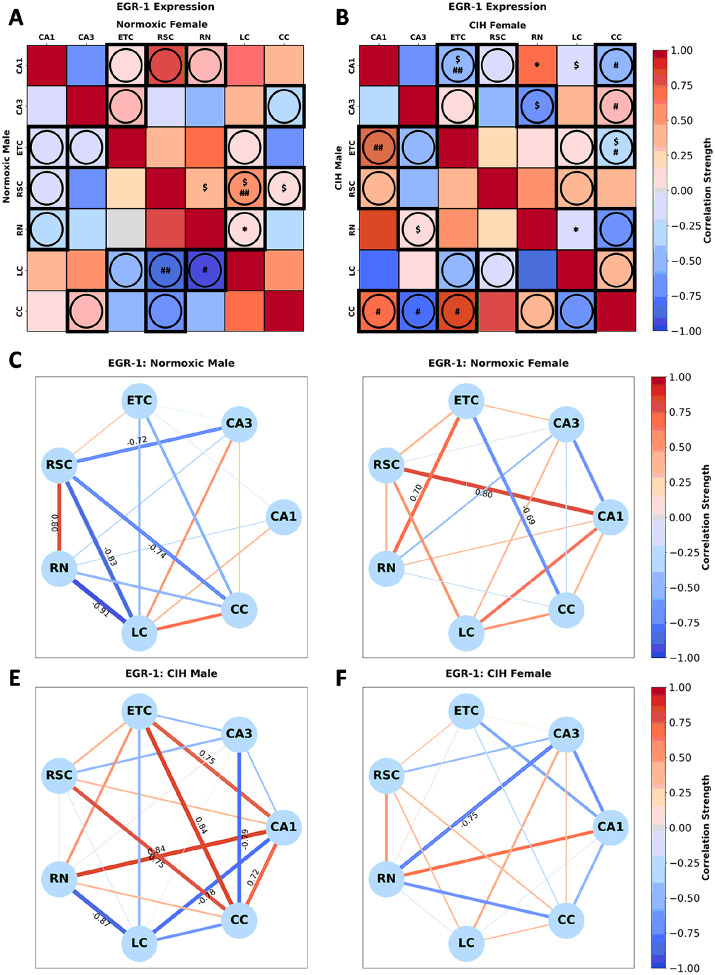
Sex and CIH differences in the inter-regional correlations of EGR-1 expression **(A)** Heatmaps of EGR-1 expression in normoxic and **(B)** and CIH males and females. **(C)** Correlation connectomes for normoxic males, **(D)**normoxic females, **(E)** CIH males, and **(F)** CIH females. Darkness of color **(A-F)** and thickness of network edges **(C-F)** represent the strength of the correlation, with correlations larger than ± 0.67 labeled. Correlations between brain regions were analyzed by Pearson correlations. Significance indicated by: $ = p≤0.10; * = p≤0.05. Correlations that are in opposite directions are indicated with a circle and green outline. Significance between sexes was determine using z-test statistics. Significance indicated by # = p≤0.10; ## = p≤0.05.

EGR-1 expression in normoxic rats (Fig. 8A) was significantly correlated in females between RSC-RN [r(n = 6) = 0.418, p = 0.056], RSC-LC [r(n = 6) = 0.547, p = 0.083], RSC-CC [r(n = 6) = 0.069, p = 0.092], and RN-LC [r(n = 6) = 0.031, p = 0.031]. No significant correlations in EGR-1 expression were observed in normoxic males. Sex differences in EGR-1 expression between RSC-LC (p = 0.028) and RN-LC (p = 0.055) were observed in normoxic rats (Fig. 8A). Conversely, EGR-1 expression in CIH rats (Fig. 8B) was significantly correlated in males between CA3-RN [r(n = 6) = 0.144, p = 0.085] and in females between CA1-ETC [r(n = 6) = −0.574, p = 0.087], CA1-RN [r(n = 6) = 0.651, p = 0.037], CA1-LC [r(n = 6) = −0.128, p = 0.067], CA3-RN [r(n = 6) = −0.752, p = 0.085], ETC-CC [r(n = 5) = −0.276, p = 0.077], RN-LC [r(n = 6) = −0.145, p = 0.024]. Sex differences in EGR-1 expression between CA1-ETC (p = 0.047), CA1-CC (p = 0.067), CA3-CC (p = 0.090), and ETC-CC (p = 0.067) were observed in CIH rats (Fig. 8B). As can be observed in Fig. 8C and Fig. 8D, multiple strong correlations in EGR-1 expression are present in normoxic males and females. A strong positive correlation between RSC-RN, and strong negative correlations between CA3-RSC, RSC-LC, RSC-CC, and RN-LC were observed in normoxic males, whereas strong positive correlations between CA1-RSC and ETC-RN and a strong negative correlation between ETC-CC were observed in normoxic females. As can be observed in Fig. 8E and 8F, CIH changes the strength and direction of many correlations in EGR-1 expression in both males and females. In CIH males, strong positive correlations between CA1-ETC, CA1-RN, CA1-CC, ETC-CC, and RSC-CC and strong negative correlations between CA1-LC, CA3-CC, and RN-LC were observed. In CIH females, only one strong negative correlation between CA3-RN was observed.

## Discussion

This is the first study to examine the effect of sex on CIH-induced protease dysregulation within brain regions associated with learning and memory. Further, no previous study has examined calpain or caspase-3 activity in females exposed to CIH. The majority of the effects of CIH on enzymatic activity were observed within the CA1 subregion of the hippocampus, ETC, and RSC (Supplemental Fig. S1).

The CA1 is highly interconnected with both the ETC and the RSC. The ETC, which serves as the primary afferent brain region to the hippocampus, connects directly to the CA1 through the corticohippocampal circuit (a.k.a. temporo-ammonic pathway) [15, 21, 100] and indirectly through the perforant pathway [25–27, 101]. The CA1 also projects directly to ETC [15, 21, 23], and the major dorsal efferent projection from the CA1 is to the RSC [15, 21, 23]. Additionally, the RSC exhibits bidirectional connections with the ETC [28, 29]. These regions are integral to the formation of multiple types of memory [18–20, 24–27], and exhibit sexually dependent CIH-induced protease dysregulation.

CIH-induced changes to protease activity and the pool of protease-cleaved fragments may result in alterations in cellular machinery that ultimately culminate in neuronal dysfunction and multiple possible cognitive impairments. Previous studies have observed that dysregulation of calpain or caspase-3 activity throughout the brain is associated with impaired attention, learning, and memory [38, 44, 45, 102–104]. Increased calpain activity has been associated with increased oxidative stress and neurodegeneration [38, 102–104]. Decreased caspase-3 activity is associated with neuronal dysfunction and decreased neurogenesis [49, 50], while increased caspase-3 activity is associated with elevated apoptosis [44, 45]. We have previously published that the CIH rats in this study exhibited impaired recollective memory, regardless of sex [66]. Importantly, the effects of CIH on protease function within the CA1, ETC, and RSC were sexually dependent and unique to each brain region. This suggests that while protease dysfunction was sex and brain region dependent, these dysfunctions may have resulted in similar cognitive impairment in both sexes.

Neuromodulatory neurotransmitters released by the RN and LC, such as serotonin and norepinephrine, are known to modify hippocampal function through direct efferent projections [30, 31] (Supplemental Fig. S1). While no effects of CIH were observed in the LC, CIH decreased caspase-3 activity in the RN of males and caspase-3 cleaved spectrin in the RN of females. Additionally, the RN was the only brain region where CIH decreased EGR-1 expression in both males and females. Decreased EGR-1 expression is associated with impaired neuronal and synaptic function [56, 57]. Impaired activity in the RN is associated with impaired cognitive function [105, 106]. Previous studies have observed CIH-dependent regulation of serotonergic activity in the vagal nerve [107, 108], though no study examining serotonergic function has been performed in the brain. This suggests that CIH may regulate serotonergic signaling from the RN, which could contribute to cognitive impairments observed in CIH.

The CC is indirectly connected with the hippocampus (Supplemental Fig. S1) and has been associated with modifying memory formation [21, 32]. However, we did not observe any effects of CIH on calpain activity, caspase-3 activity, calpain cleaved spectrin, or caspase-3 spectrin in the CC, regardless of sex. Though we previously reported that the CIH females from this study exhibited impaired ne motor function [66], it is unlikely these behavioral differences were due to changes within the CC. Neither spatial navigation nor gross motor impairments were impaired [66], which are behaviors more commonly associated with the CC [32].

A connectome analysis was used to determine how CIH influenced calpain activity, capase-3 activity, and EGR-1 expression among the different regions that may contribute to cognitive function. In normoxic male rats, calpain activity in RN had strong positive relationships with CA3 and CA1. This contrasted with normoxic female rats, as no correlations between these regions were observed. In female rats, calpain activity in CA1 did not share strong positive relationships with any single region. Instead, calpain activity in the female CA3 was positively related to calpain activity in the LC while calpain activity in CA1 was still positively related to RN. Exposure to CIH changed these relationships in the brains of both the males and the females. In males, the strength of the relationship between RN and dorsal hippocampus decreased dramatically and the relationship between CA1 and CA3 changed from a weak positive correlation to a strong negative correlation. CIH exposure in female rats shifted the relationships in calpain activity of the dorsal hippocampus away from the LC to the RN creating a pattern similar to that observed in normoxic males. Calpain has several isoforms that can support signaling pathways that can promote either neuroplasticity or cell death [42]. The functional significance of these sex and regionally specific changes in calpain activity are likely dependent on which isoform is expressed in these regions. Future studies investigating the concentrations of the isoforms of calpain in these brain regions are necessary to fully appreciate the mechanism of CIH in these brain regions. However, it is interesting to note that CIH has been shown to have either neuroprotective or neurodegenerative effects depending on the protocol used [109].

The connectome analysis of caspase-3 activity also showed sex-based differences that were regionally specific. In male rats, all the regions that were examined exhibited strong positive correlations except the RSC, and CIH exposure reduced or reversed these relationships with a few notable exceptions related to the RSC and the ETC. There were a few strong positive correlations in normoxic female rats involving the RSC and the RN and ETC and the CA1 and CC. The positive correlation for caspase-3 activity between the RSC and ETC did not appear to be affected by CIH in female rats while all other aspects of the connectome were reduced. The differences in caspase-3 activity between males and females could reflect basal sex-based differences required to maintain synaptic homeostasis [49] or synaptic plasticity [50, 51]. The generalized decreased caspase-3 activity associated with CIH in both males and females is consistent with a previous study using hypoxic preconditioning [39]. In the Lv et al. study, decreased active caspase-3 was associated with less propofol-induced neural apoptosis. The CIH protocol used in the current study is associated with cognitive impairment [66–69] and the decreases in caspase-3 activity seen following CIH may contribute to these behavioral effects.

The connectome for EGR-1 expression showed different outcomes in male and female rats exposed to CIH. In normoxic male rats, the strongest relationships were negative correlations involving the RSC with CA3, CC, and LC. The LC also demonstrated a strong negative relationship for EGR-1 expression with the RN. The only strong positive correlation for EGR-1 expression in normoxic males was between the RSC and the RN. Following CIH, strong positive correlations emerged between the RSC and CC, ETC and CC, CA1 and CC along with positive correlations between CA1 and the RN and the ETC. Two new negative correlations in EGR-1 activity are following CIH: between the LC and CA1 and between the CC and CA3. Overall, these patterns appear to represent low levels of activity-dependent EGR-1 mediated genes in these regions in normoxic males that are altered to higher levels of EGR-1 expression and presumably changes in the expression of EGR-1 sensitive genes following exposure to CIH. In normoxic female rats, EGR-1 expression showed strong positive correlations between the RSC and CA1 and the RN and ETC and a strong negative correlation between the ETC and the CC. In female rats exposed to CIH, the strong positive correlations that were present in normoxic females were decreased leaving the negative correlation between the RN and CA3 as the remaining strong correlation. In this respect, CIH differentially affected the EGR-1 connectome in males and females. CIH increased positive correlations for EGR-1 in males while it decreased the number of positive correlations in females.

Similar to our previous studies, we observed CIH-decreased calpain activity in the ETC, no effects of CIH on calpain activity in the male dorsal hippocampus, and no effect of CIH on caspase-3 activity in male dorsal hippocampus or ETC [75]. Although our caspase-3 results are inconsistent with most studies that utilized CIH in rodent models and identified CIH-induced caspase-3 activity and apoptosis in the hippocampus [70–73], these studies use a more severe hypoxia protocol. Our studies have utilized a CIH exposure protocol comparable to mild OSA in humans [79, 81]. Studies which have observed elevated caspase-3 activity [70–73] performed CIH protocols comparable to moderate or severe OSA [79, 81]. As the severity of OSA is positively correlated with the severity of cognitive impairments [81], it is plausible that protease dysregulation would follow the same pattern. Additionally, we have previously reported rat strain-specific responses to CIH [79], which may explain why our current experiment in Sprague Dawley rats does not corroborate with previous studies in other strains.

This is the first study to examine sex differences in the activity of calpain and caspase-3. We observed sex differences in protease activity in the RSC and calpain cleaved spectrin in the ETC and RSC with lower activity and protein expression observed in the females. Importantly, the physiological and the behavioral effects of these sex differences are currently unknown. Previous studies, which examined EGR-1 expression in multiple brain regions, have identified sex differences in the activity of EGR-1 following either learning or aversive stimuli [59–62]. These previous studies are broadly consistent with our findings, as they observed no sex differences in the CA1 [59–61], CA3 [59] or greater activity in the CA1 [62] and CA3 of females [60, 61]. Importantly, the studies that observed sex differences in EGR-1 activity of these brain regions, which were not observed in our experiments, found that the observed sex differences were dependent on several confounding factors. These factors included the use of aversive stimuli to induce EGR-1 activity [62], the learning strategies employed by the rats [60, 61], and reproductive estrous status of the females [61]. No aversive stimuli were utilized in our behavioral experiments; therefore, it is unlikely we would observe the same effects in our rats. Additionally, we did not examine either the learning strategies or estrous status of our rats.

### Limitations

This study has multiple strengths such as the examination of multiple brain regions associated with cognition and molecular analysis of proteins relevant to neuronal function. However, future studies will need to be conducted as there are limitations to this study. For example, we were not able to examine the contributions of gonadal hormones to these effects. Estradiol is reported to alter brain connectivity [110–112], and could contribute to both sex differences and CIH effects. However, we have previously published that no differences in circulating estradiol were observed in these rats, regardless of CIH or sex [66]. The brain regions we examined do not represent an exhaustive list of brain regions functionally related to the dorsal hippocampus. Additional regions not examined in this study but associated with the hippocampus, such as the thalamus, hypothalamus, or amygdala, may have been affected by CIH [20, 58, 67, 113, 114]. As we observed impaired recollective memory in these rats [66], we focused on brain regions related to cognitive function. Future studies will need to assess if the CIH-induced dysregulation of protease activity in the CA1 and connected cortices is associated with changes in the fiber tracts that connect these regions. Axonal or dendritic morphological or functional changes could be identified through the use of immunohistochemistry or light microscopy [115]. Further, the downstream consequences of protease dysregulation need to be assessed. We were unable to examine these downstream signaling cascades due to limited protein concentration in our samples. Therefore, future studies focused on the regulatory mechanisms for protease activity in CIH and downstream signaling pathways associated with protease activity and neuronal activation are warranted. Specifically, a better resolution of the association between the activation of cytosolic proteases and neuronal activation is needed to better understand how these molecular events are triggered, as well as the consequences of their activation, in specific brain regions of males and females.

### Perspectives and Significance

Our findings reveal brain region- and sex-specific vulnerabilities to mild CIH. In our previous study in these rats, we observed impairments in novel object behaviors in both males and females [66]. Novel object behavior is associated with recollective and working memory [116]. All brain regions where protease activity was dysregulated by CIH in this study are associated with memory function [18–20, 24–27, 105, 106]. However, male and female brains exhibited region-specific CIH-induced protease dysfunction. As our study models mild OSA, and protease dysregulation was more commonly induced by CIH in the female brains, this may indicate protease function is a sexually dependent mechanism associated with OSA-induced cognitive impairment. Our connectomes also revealed CIH induced sex-specific shifts in correlational patterns. CIH alterations in EGR-1 expression correlated between male brain regions may indicated increased excitatory activity that was not observed in females. This suggests latent sex differences are involved in CIH-induced cognitive impairments in male and female rats, whereby sex differences in CIH-induced protease dysfunction and brain connectivity underlie the behavioral phenotype of cognitive impairment. Further investigation into these sex and brain-region specific vulnerabilities to CIH is warranted.

## Figures and Tables

**Table 1:** Protease activity and cleavage products in the hippocampus and associated brain regions

		Male		Female	
		Normoxia	CIH	Normoxia	CIH
**CA3**	Calpain Activity	108.96 ± 54.12	113.73 ± 29.26	138.61 ± 66.66	135.91 ± 80.75
	Caspase-3 Activity	30.02 ± 21.12	37.33 ± 19.92	45.87 ± 31.79	32.38 ± 18.37
	Calpain Cleaved Spectrin	113.05 ± 84.41	132.23 ± 93.09	108.40 ± 43.42	140.83 ± 59.18
	Caspase-3 Cleaved Spectrin	27.48 ± 22.37	42.98 ± 40.94	29.65 ± 8.46	37.81 ± 24.86
**CC**	Calpain Activity	38.96 ± 16.30	48.31 ± 15.88	45.90 ± 16.12	35.06 ± 9.17
	Caspase-3 Activity	10.04 ± 6.14	13.66 ± 9.01	12.25 ± 6.48	14.02 ± 8.13
	Calpain Cleaved Spectrin	38.39 ± 19.86	43.37 ± 15.80	54.20 ± 20.17	38.98 ± 17.08
	Caspase-3 Cleaved Spectrin	9.97 ± 6.62	14.25 ± 7.71	13.13 ± 5.16	13.43 ± 5.66
**LC**	Calpain Activity	54.79 ± 28.30	46.16 ± 28.76	123.57 ± 101.08	47.05 ± 21.70
	Calpain Cleaved Spectrin	47.32 ± 36.33	72.45 ± 40.40	29.85 ± 15.75	105.24 ± 98.68

Calpain and caspase-3 activity represent % of protease cleaved spectrin to uncleaved spectrin (250 kD). Calpain cleaved spectrin (150 kD) and caspase-3 cleaved spectrin (120 kD) expression normalized to β-actin. All values presented as mean ± SD. Analyzed by 2-way ANOVA with Fisher’s LSD multiple comparisons tests. *CC: Cerebellar cortex; CIH: Chronic intermittent hypoxia; LC: Locus coeruleus*.

**Table 2:** EGR-1 expression in the hippocampus and associated brain regions

	Male		Female	
	Normoxia	CIH	Normoxia	CIH
**CA3**	61.89 ± 26.77	112.78 ± 97.59	79.09 ± 25.68	94.75 ± 50.57
**CC**	50.00 ± 18.35	65.88 ± 7.59	75.58 ± 49.57	56.76 ± 10.87
**LC**	74.87 ± 32.86	239.05 ± 269.37	186.91 ± 61.62	164.74 ± 149.10

EGR-1 expression normalized to β-actin. All values presented as mean ± SD. Analyzed by 2-way ANOVA with Fisher’s LSD multiple comparisons tests*. CC: Cerebellar cortex; CIH: Chronic intermittent hypoxia; LC: Locus coeruleus*.

## Data Availability

The datasets used and/or analyzed during the current study are available from the corresponding author upon reasonable request.

## Highlights

- This is the first study to examine the role of sex in CIH-induced protease dysregulation in the rat brain and to examine sex differences in calpain and caspase-3 activity in rats.
- CIH predominantly dysregulated protease activity in memory-associated brain regions of female rats, which may indicate a sex-specific vulnerability to mild OSA.
- CIH only impaired EGR-1 expression in the raphe nucleus, regardless of sex, with no effect observed in other brain regions.
- CIH altered the sex-specific correlational relationships of protease activity and neuronal activation between brain regions.
- CIH increased excitatory neuronal activity between brain regions in males but not females.
